# Patient-Centered Outcomes of Microfragmented Adipose Tissue Treatments of Knee Osteoarthritis: An Observational, Intention-to-Treat Study at Twelve Months

**DOI:** 10.1155/2020/8881405

**Published:** 2020-08-04

**Authors:** Nima Heidari, Ali Noorani, Mark Slevin, Angela Cullen, Laura Stark, Stefano Olgiati, Alberto Zerbi, Adrian Wilson

**Affiliations:** ^1^The Regenerative Clinic, 18-22 Queen Anne Street, London, UK; ^2^Department of Life Sciences, Manchester Metropolitan University, UK; ^3^Dept. of Morphology, Surgery and Experimental Medicine, Faculty of Medicine, University of Ferrara, 44121 Ferrara, Italy; ^4^University of Milano, Dept. of Radiology, 20100 Milano, Italy

## Abstract

**Introduction:**

Microfragmented adipose tissue (MFAT) has been shown to benefit osteoarthritic patients by reducing pain and supporting tissue regeneration through a mesenchymal stem cell (MSC)-related paracrine mechanism. This observational study of 110 knees assessed patient-centered outcomes of pain, functionality, and quality of life, analyzing their variation at twelve months following one ultrasound-guided intra-articular injection of autologous MFAT for the treatment of knee osteoarthritis (KOA).

**Method:**

Inclusion criteria were as follows: VAS >50, and the presence of KOA as diagnosed on X-ray and MRI. Exclusion criteria included the following: recent injury (<3 months) of the symptomatic knee, intra-articular steroid injections performed within the last three months, and hyaluronic acid injections prior to this treatment. Changes in VAS, OKS, and EQ-5D were scored at baseline and twelve months following a single intra-articular injection of autologous MFAT. Score variation was analyzed utilizing a nonparametric paired samples Wilcoxon test. The statistical analysis is reproducible with Open Access statistical software R (version 4.0.0 or higher). The study was carried out with full patient consent, in a private practice setting.

**Results:**

Median VAS (pain) improved from 70 (IQR 20) to 30 (IQR 58) (*p* < 0.001); median OKS (function) improved from 25 (IQR 11) to 33.5 (IQR 16) (*p* < 0.001); and median EQ-5D (quality of life) improved from 0.62 (IQR 0.41) to 0.69 (IQR 0.28) (*p* < 0.001). No adverse events were reported during the intraoperative, recovery, or postoperative periods.

**Conclusions:**

For patients with all grades of knee osteoarthritis who were treated with intra-articular injections of MFAT, statistically significant improvements in pain, function, and quality of life were reported. Although further research is warranted, the results are encouraging and suggest a positive role for intra-articular injection of MFAT as a treatment for knee osteoarthritis.

## 1. Introduction

Osteoarthritis (OA), the most prevalent form of arthritis, is characterized by chronic progressive degeneration and alteration of hyaline articular cartilage, subchondral bone, ligaments, capsule, synovium, and periarticular muscles [1]. The condition currently presents a substantial global socioeconomic burden and remains a leading cause of disability, particularly among the elderly population [2].

With an ageing population, this is a growing problem and is set to become the fourth leading cause of disability by the end of 2020 [3, 4]. In the UK, approximately one in seven people suffer with arthritis. This equates to ten million individuals. It is a complex and multifactorial disease involving genetic, environmental, and mechanical causes [5]. The disease can be classified into one of two groups depending upon its aetiology: primary (idiopathic or nontraumatic) and secondary (a result of trauma or mechanical malalignment) [6].

Advancements in regenerative medicine over the last decade have revealed the potential for mesenchymal stem cells (MSCs) to be used as a powerful therapeutic tool against tissue damage and degeneration [7–9]. Mesenchymal stem cells (MSCs), more recently termed Medicinal Signaling Cells [10], have shown promise as a standalone treatment for OA, or as an adjunct to traditional surgical correction of the mechanical environment. Since their discovery by Friedenstein in the late 1960s [11], extensive research has been implemented in an effort to exploit these cells for their true therapeutic potential.

The cells arise from pericytes that are found naturally occurring around the vasculature in various tissues throughout the body. Upon disruption of the stromal vascular fraction of tissue, a significant proportion of pericytes lift from the surface of the vessels and contribute to the tissue homeostatic response as the source of adipose-derived stem cells-MSCs [12, 13].

These MSCs respond by producing bioactive signaling molecules which act in a paracrine fashion to exhibit immunomodulatory, antimicrobial, angiogenic, and trophic/regenerative effects on tissue [5, 14]. This ultimately results in localized and tissue-specific change that encourages regeneration and healing [10].

Sources for MSCs include bone marrow, placenta, and dental pulp [15]. The use of such cells in biological therapy has always been hindered as they are challenging to obtain in large enough quantities to provide significant clinical benefit. Additionally, the extraction of such cells from bone marrow is a painful procedure, and a 1 ml sample provides only 0.01% of MSCs. Conversely, adipose tissue has been recognized as a reliable and potent source for MSCs. One MSC can be obtained per 100 adipose cells, in contrast to 1 MSC for every 100,000 bone marrow cells [16–18]. A promising feature of adipose-derived MSCs is in their localization as they represent 10-30% of normal body weight and produce a concentration of 5,000 cells per gram of tissue obtained [5, 19]. The use of adipose-derived MSCs is associated with minimal side effects arising and previous studies have shown no complications relating to malignancy or cancer [20]. It has also been suggested that these adipose-derived MSCs have multilineage potential and possess the same regenerative capacity as those derived from other tissues. Furthermore, the cells are not adversely influenced by the age of the patient; a factor that is very beneficial for an elderly population [21].

Given that arthritis is such a prevalent condition, especially among an ageing population [22], it is vital to identify the long-term efficacy of therapies that utilize the regenerative power of adipose-derived MSCs, and whether they can potentially prevent replacement surgery or delay it for as long as possible. Furthermore, it is important to identify whether treatment with adipose-derived MSCs, or as in this case, MFAT-containing MSCs (originating from the adipose blood vessels as pericytes, and that are released and primed during the extraction process through sheer stress and microfiltration of the fat) [23, 24] would be beneficial in providing an optimal biological environment for healing when used in conjunction with surgery [25]. Their use for the treatment of knee osteoarthritis (KOA) has produced very encouraging results [26–28]. This case series aims to assess the response of our patient cohort over a one-year period following a routine single ultrasound-guided intra-articular injection of MFAT for all grades of KOA.

## 2. Method

The study was conducted in accordance with the principles of Good Clinical Practice (NIHR) and the General Medical Council (GMC) guidelines on research, patient consent to research and future publication, as well as adhering to and in accordance with the Declaration of Helsinki. The study was carried out in a private practice setting.

This observational, intention-to-treat study included the complete sample of 110 patients who agreed to be scored for pain (Visual Analogue Scale—VAS), function (Oxford Knee Score—OKS), and quality of life (EuroQol-5D—EQ-5D) at baseline regardless of subsequent changes to adherence or status during follow-up. All patients attended the private clinics of the authors (AW, NH) complaining of knee pain following a diagnosis of knee osteoarthritis.

Patients underwent clinical review and examination by an orthopaedic surgeon. The preoperative assessments included evaluation of imaging (X-ray in all cases and MRI in some) where the KOA was graded using the Kellgren & Lawrence (KL) grading system.

Inclusion criteria included VAS >50, no deformity greater than ten degrees of varus or valgus, and the presence of KOA as diagnosed on X-ray and/or MRI. Exclusion criteria included recent injury (<3 months) of the symptomatic knee, infectious joint disease, malignancy, pregnancy, anticoagulation or thrombocytopenia, coagulation disorder, and intra-articular steroid injections performed within the last three months. None of our patients had hyaluronic acid injections prior to this treatment.

The patients were informed of all possible options for treating their KOA including conservative means as well as injections of a number of substances including steroids, hyaluronic acid, platelet-rich plasma, and microfragmented adipose tissue. They also had surgical options detailed to them including osteotomy, partial and total knee replacement.

By study design, the paired samples have been selected and not randomized, so we could not assume a Gaussian distribution. For this reason, scores variation has been analyzed utilizing a nonparametric paired samples Wilcoxon test to assess statistically significant changes in the VAS, OKS, and EQ-5D scores before and after treatment at twelve months. For the same reason, summary statistics report median and interquartile ranges (IQR).

**Figure 1:**
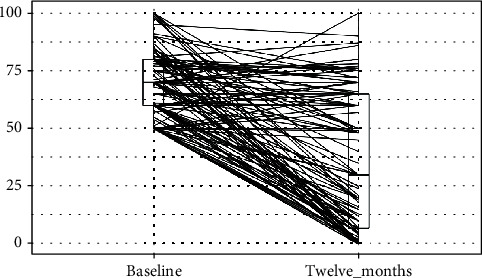
VAS scores at baseline and at twelve months follow-up. *y*-axis: Visual Analogue Scale (VAS) for pain value; boxplot showing L-estimators: maximum (100), minimum (50), median (70), and interquartile range (20); outliers plotted as individual points at baseline and maximum (100), minimum (0), median (30), and interquartile range (58); outliers plotted as individual points at 1 year follow-up. *x*-axis: Visual Analogue Scale (VAS) for pain at preoperative baseline and at twelve months follow-up. Connecting lines: heuristic visualization of single-patient trajectories of variation of Visual Analogue Scale (VAS) value for pain between preoperative baseline and twelve months follow-up; plotted with R (version 4.0.0 or higher; libraries ggpubr and PairedData). Source: Authors' Data and reproducible statistical analysis with Open Access statistical software R (version 4.0.0 or higher).

**Figure 2:**
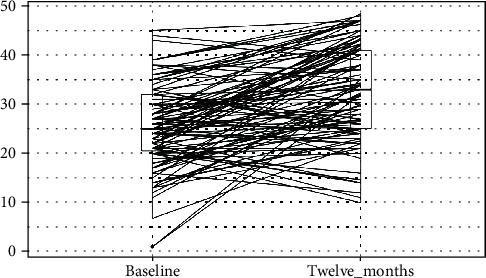
Oxford Knee Scores at baseline and at twelve months follow-up. *y*-axis: Oxford Knee Score (OKS) for function value; boxplot showing L-estimators: maximum (45), minimum (1), median (25), and interquartile range (11); outliers plotted as individual points at baseline and maximum (47), minimum (10), median (33), and interquartile range (16); outliers plotted as individual points at 1 year follow-up. *x*-axis: Oxford Knee Score (OKS) for function at preoperative baseline and at twelve months follow-up. Connecting lines: heuristic visualization of single-patient trajectories of variation of Oxford Knee Score (OKS) value for function between preoperative baseline and twelve months follow-up; plotted with R (version 4.0.0 or higher; libraries ggpubr and PairedData). Source: Authors' Data and reproducible statistical analysis with Open Access statistical software R (version 4.0.0 or higher).

**Figure 3:**
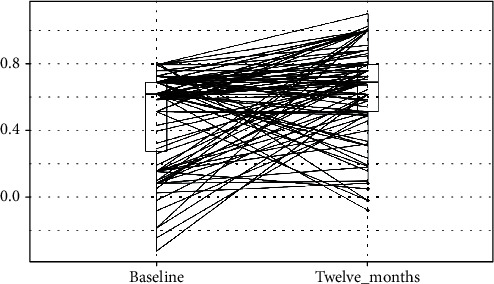
EQ-5D Scores at baseline and at twelve months follow-up. *y*-axis: EQ-5D for quality of life score value; boxplot showing L-estimators: maximum (0.812), minimum (-0.319), median (0.62), and interquartile range (0.41); outliers plotted as individual points at baseline and maximum (1), minimum (-0.074), median (0.69), and interquartile range (0.28); Outliers plotted as individual points at 1 year follow-up. *x*-axis: EQ-5D for quality of life score at preoperative baseline and at twelve months follow-up. Connecting lines: heuristic visualization of single-patient trajectories of variation of EQ-5D for quality of life score value for function between preoperative baseline and twelve months follow-up; plotted with R (version 4.0.0 or higher; libraries ggpubr and PairedData). Source: Authors' Data and reproducible statistical analysis with Open Access statistical software R (version 4.0.0 or higher).

**Figure 4:**
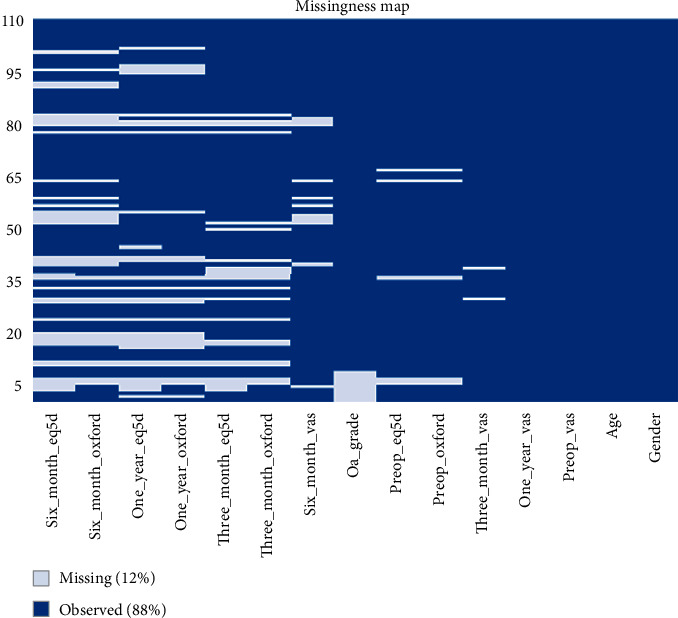
Missingness map. *x*-axis: outcome variables: gender, age, Visual Analogue Scale (VAS) for pain, Oxford Knee Score (OKS) for function, and EQ5D for quality of life at preoperative baseline and three, six, and twelve months follow-up. *y*-axis: data points missing at random (12%) due to patients lost-to-follow-up: missing data have been estimated probabilistically with uncertainty using the statistical software package Amelia v1.7.6 or higher. The estimation procedure is replicable and reproducible with Open Access statistical software R (4.0.0 or higher). The missingness map shows the missing data fields in our dataset. All patients have a preprocedure and 1 year VAS for pain as seen on the right-hand column of the plot. The left-hand column then the missing OKS and EQ5D.

Summary statistics, statistical analysis, and statistical significance testing are reproducible with Open Access statistical software R (version 4.0.0 or higher; R function Wilcox test). Figures 1, 2, and 3 have been generated automatically from data by Open Access statistical software R (version 4.0.0 or higher; libraries ggpubr and Paired-Data); Table 1 summarizes the change in the median VAS, OKS, and EQ-5D scores at 12 months from baseline according to the OA grading. Data points are missing at random (12%) due to patients lost-to-follow-up; missing data have been estimated probabilistically with uncertainty (unbiased) using the statistical software package Amelia v1.7.6 or higher. The estimation procedure is replicable and reproducible with Open Access statistical software R (4.0.0 or higher). The missingness map is visualized in Figure 4.

### 2.1. Patients

The series of 110 cases was comprised of 60 male and 50 female patients, with ages ranging from 42 to 94. Most of the patients had advanced KOA with 80% having a Kellgren-Lawrence (KL) grade of III or IV. 95 (96.4%) of the patients had idiopathic KOA, and 4 (3.6%) had posttraumatic KOA. (Table 2).

One patient with posttraumatic OA had an injury as a child, and the other 3 had a combination of meniscal and ACL injuries that then lead to KOA.

### 2.2. Source: Authors' Data

Full and informed consent was undertaken for each part of the procedure including sedation, lipoasipration, and image-guided intra-articular injection. All procedures were performed in an operating theatre as a day case, and patients were discharged approximately three hours following the completion of the procedure.

### 2.3. Harvesting the Adipose Tissue

The patient was placed under a sedation administered by an anaesthetist. A small incision was made to insert a 17G blunt cannula (connected to a luer-lock 60-cc syringe), and Klein sterile solution (containing saline, Lignocaine, and epinephrine) injected into the subcutaneous fat. Approximately, 150-200 ml of this solution was injected in 50 ml aliquots into the lower abdominal area. Adipose tissue (approximately 50 ml) was then harvested manually via a 13G blunt cannula (connected to a Vaclock 20 ml syringe), by a consultant plastic surgeon, experienced in this procedure. The area of fat harvest was tailored to the body habitus of each patient, (normally lower abdomen or flank areas) with the patient in a supine position. An abdominal binder was then applied to the adipose tissue harvest site.

### 2.4. Processing and Injecting the Lipoaspirate

The lipoaspirate was processed using the Lipogems® system [29]. This is a disposable and single-use device. The lipoaspirate is introduced in a closed and aseptic manner into the low-pressure, full immersion, transparent plastic cylindrical container through stainless steel wire mesh. The device is prefilled with saline. Within the container, there are stainless steel ball bearings that work to mechanically fragment the fat, progressively reducing the size of the clusters of adipose tissue (from spheroidal clusters with a diameter of 1–3.5 mm to clusters of 0.2–0.8 mm) through mechanical agitation of the chamber much like a cocktail shaker. The chamber is flushed with saline to wash out impurities (e.g., oil, blood, and proinflammatory debris). The resulting product is then filtered through a 500-micron filter. This process takes approximately 20 minutes. A single 6-8 ml of this refined product was then injected directly into the knee joint under ultrasound guidance. This point of care device allows the procedure from lipoaspiration to the injection of the microfragmented fat to take place within the same sitting mitigating the need for any repeat visits from the patient.

### 2.5. Postoperative Care

All patients were provided with a pack which included analgesia (paracetamol and codeine), as well as a printed physiotherapy protocol. We advised our patients to avoid nonsteroidal anti-inflammatory drugs. Other nonpharmaceutical means of pain control such as rest and the use of warm and cold packs were advocated. Following discharge, outpatient physiotherapy sessions were scheduled for each patient. Patients were allowed to bear weight on their joint postprocedure; however, they were instructed to avoid any strenuous or high-impact activities for two weeks. Chemical thromboprophylaxis was not prescribed.

### 2.6. Multiple Outcome Measurements

Outcomes were measured using the Visual Analogue Scale (VAS) for pain, the Oxford Knee Score (OKS) for function, and the EQ5D for quality of life. All patients completed these questionnaires before treatment, and at three months, six months, and one year following treatment. Our analysis in this report includes the 12 months data.

VAS [30] is a validated measurement system that allows participants to measure their pain intensity along a continuous scale of values that otherwise cannot clearly be measured. Participants are presented with a horizontal line that is anchored by two extremes, between 0 and 100 (0 = no pain, 100 = worst pain), and are asked to place a point along the VAS line at the point that would represent their current level of pain.

OKS [31] comprised of 12 questions that were scored 0-4 with 0 being severe and 4 being none, covering pain and function of the knee. The best outcome is a score of 48, and the worst score possible is 0.

EuroQol-5 Dimension [32] is a standardized instrument developed by the EuroQol Group in order to measure the health-related quality of life in a wide range of medical conditions. Five dimensions are measured in the respondent: mobility, self-care, usual activity, pain, and anxiety/depression. Scores were given between 1 and -1; this was recorded down with 1 being associated with a better quality of life whilst -1 the opposite.

## 3. Results

### 3.1. Summary Results

Median VAS (pain) improved from 70 (IQR 20) at baseline to 30 (IQR 58) at twelve months (*p* < 0.001); median OKS (function) improved from 25 (IQR 11) to 33.5 (IQR 16) (*p* < 0.001); and median EQ-5D (quality of life) improved from 0.62 (IQR 0.41) to 0.69 (IQR 0.28) (*p* < 0.001). No adverse events were reported during the intraoperative, recovery, or postoperative periods.

**Figure 5:**
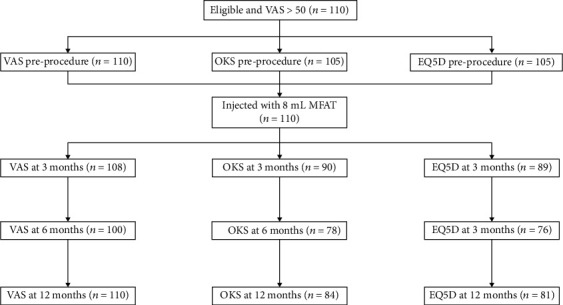
Study flow diagram depicts the touch points including the preprocedure assessment, injection of the knee, and subsequent follow-up and attrition in the collection of outcomes.

Summary results are presented in Table 3 and Figures 1, 2, and 3.  Figure 5 shows the study flow diagram with the data collection points of the study and attrition rate for collected scores. The missingness map (Figure 4) presents where scores have not been provided by the patients.

In Table 1, the variation of median VAS, OKS, and EQ-5D between baseline and twelve months follow-up has been grouped according to the grade of KOA. This shows a general trend in all severity of KOA towards improvement, but these data are not suitable for detailed subgroup analysis and statistical significance testing as grades 1 and 2 only represented 20% of the total sample.

## 4. Discussion

We report here the results of treating degenerative arthritis of the knee with autologous microfragmented adipose tissue. We found that 81% of our patients experienced a reduction in their pain and a concomitant improvement in their function with a single injection of MFAT. Median VAS, OKS, and EQ-5D between baseline and twelve months follow-up improved for all grades of KOA (Table 1). This shows a general trend in all severity of KOA towards improvement, but these data are not suitable for detailed subgroup analysis and statistical significance testing as grades 1 and 2 only represent 20% of the total sample size. The risks of this procedure are low and the possibility of reverting to more interventional approaches in those who do not improve remains. Of note is that higher grades of arthritis (KL grades III and IV) demonstrated an improvement in pain and function.

Knee OA is a debilitating condition that affects a significant proportion of the population in all nationalities. Current solutions include the correction of deformities to preserve the knee or otherwise joint sacrificing procedures such as total or partial replacement. These surgical options carry risk, and many individuals seek nonsurgical solutions and are not willing to consider surgery until these have been exhausted.

To this end, a growing number of clinicians are using biologics such as Platelet Rich Plasma and cell-based therapies to control pain in arthritis and delay the need for surgical intervention. Among these therapies being investigated is the use of microfragmented adipose tissue. A search of clinicaltrials.gov revealed a number of ongoing studies assessing the effects of microfragmented adipose tissue for KOA.

In our study, we noted a very low number of adverse events and complications with pain at the harvest and injections sites. This experience is mirrored in the literature with a prospective study of 1524 patients, many with significant medical comorbidities, who received stromal vascular fraction procedures. At long term follow-up (22 to 64 months), 98% reported no adverse events and 0.72% reported a new cancer diagnosis [33]. In a systematic review and meta-analysis of 36 clinical trials of both autologous and allogeneic mesenchymal stem cells harvested from several tissue sources, there was no evidence of increased acute toxicity, organ system complications, infection, death, or malignancy [34].

More recently a number of clinical studies have demonstrated the safety and efficacy of microfragmented fat for the treatment of KOA. Russo et al. [35] reported on 30 patients who were treated with microfragmented fat as an adjuvant for the surgical treatment of diffuse degenerative chondral lesions, with a follow-up of three years. 22 required no further treatment, and no adverse events were reported. They also noted that the improvements in Tegner-Lysholm Knee, VAS, IKDC-subjective, and total KOOS scores observed at one year were maintained at the three-year mark.

Panni et al. [27] reported similar results with 52 patients with early KOA, who received arthroscopic debridement followed by injection of microfragmented fat. At final follow-up, 96.2% of patients expressed satisfaction and reported good or excellent improvements in function and/or pain. Hudetz et al. [26] conducted a study where 20 patients with KOA were treated with a single intra-articular injection of microfragmented fat. He noted that 17 (85%) showed a substantial pattern of KOOS and WOMAC improvement, significant in all accounts.

### 4.1. Study Limitations

Our experience has mirrored that of many other colleagues regarding the use of MFAT in KOA. This treatment offers a minimally invasive and nonsurgical method for the treatment of KOA. The main limitation of this study is the absence of a control group in our sample. This self-selected group did not want to have major surgery when they came to our clinic and were treated with an ultrasound-guided single injection of MFAT. We included all grades of arthritis but excluded those with deformity greater than ten degrees. It can be argued that this represents a heterogeneous group of disease. Combining the age range of our cohort (42 to 94) as well as the severity of their conditions (KL grade I-IV) makes for many variables and thus makes subgroup analysis difficult. However, this is a pragmatic representation of our clinical practice, and the highly statistically significant improvement of pain, function, and quality of life cannot be ignored.

The missingness map and study flow diagrams show an attrition rate of 12% in our data collection. Responder fatigue is a well-documented phenomenon and may introduce bias [36].

## 5. Conclusions

The benefit of this treatment to the individual is in the mitigation of complications and the associated recovery from surgical intervention. The aim of any intervention is the reduction in pain and improvement in function with a resultant betterment of quality of life. The hope is that this will then be reflected in the overall healthcare costs at a population level. Despite the limitations detailed above, our study represents an incremental step in defining the place for biologic treatments in degenerative joint disease. These findings are mirrored in a number of other studies [26–28]. What remains unclear is whether true restoration of the cartilage occurs or indeed if this is necessary for the clinical effects seen.

The authors caution that the retrospective nature of the study and the small number of patients make it impossible to draw definitive conclusions. The findings need to be validated with a randomized controlled trial, conducted over a longer period to check for long-term outcomes, to establish more clearly the stage of the disease best suited for treatment with biologics in order to maximize patient-centered efficacy.

## Figures and Tables

**Table 1:** Variation of median VAS, OKS, and EQ-5D between baseline and 12 months follow-up grouped by OA Grade.

oa_grade	Count	VAS	OKS	EQ-5D
1	1	↑-48	↑2	↑0.2
2	12	↑-54	↑5.5	↑0.14
3	20	↑-28.5	↑3.5	0
4	68	↑-32.5	↑7	↑0.07
NA	9	↑-17	↑4	↓-0.03

Legend: variation of median VAS, OKS, and EQ-5D between baseline and twelve months follow-up has been grouped according to the grade of KOA. VAS improvement is a reduction in score plotted in green and arrow-up. All grades of OA show an improvement in pain as evidence by a reduction in the median VAS score. OKS also improved in all grades of OA with the most gains being made by those with the grade 4 group. EQ-5D improvement is an increase in score plotted in green and arrow-up, deterioration in red, and invariance in black. This shows a general trend in all severity of KOA towards improvement, but these data are not suitable for detailed subgroup analysis and statistical significance testing as grades 1 and 2 only represent 20% of the total group.

Source: Authors' Data and reproducible statistical analysis with Open Access statistical software R (version 4.0.0 or higher).

**Table 2:** Patients' characteristics.

Characteristics	Number of patients (total = 110)	Percentage of patients (%)
Sex		
Male	60	54.5
Female	50	45.5
Age (years)		
Under 50	6	5.5
50-59	26	23.6
60-69	30	27.3
70-79	34	30.9
Over 80	14	12.7
Kellgren-Lawrence grade		
I	1	0.9
II	12	10.9
III	20	18.2
IV	68	61.8
Missing data	9	8.2
Aetiology of OA		
Idiopathic	106	96.4
Posttraumatic	4	3.6

**Table 3:** Summary results.

Parameter	Assessment	Median score	IQR score	Nonparametric paired samples Wilcoxon test *p* value
VAS	Pre-op	70	20	*p* < 0.001
	1 year	30	58	
OKS	Pre-op	25	11	*p* < 0.001
	1 year	33	16	
EQ-5D	Pre-op	0.62	0.41	*p* < 0.001
	1 year	0.69	0.28	

Summary of the results showing the median values and Interquartile range (IQR) of Visual Analogue Scale for pain (VAS), Oxford Knee Score (OKS), and EuroQuol 5D (EQ-5D) at baseline and at 1 year follow-up. A statistically significant improvement in all parameters is demonstrated with *p* < 0.001.

Source: Authors' Data and reproducible statistical analysis with Open Access statistical software R (version 4.0.0 or higher).

## Data Availability

All data is available upon request
